# Sex differences in facial emotion recognition across varying expression intensity levels from videos

**DOI:** 10.1371/journal.pone.0190634

**Published:** 2018-01-02

**Authors:** Tanja S. H. Wingenbach, Chris Ashwin, Mark Brosnan

**Affiliations:** Department of Psychology, University of Bath, Bath, Somerset, United Kingdom; Bournemouth University, UNITED KINGDOM

## Abstract

There has been much research on sex differences in the ability to recognise facial expressions of emotions, with results generally showing a female advantage in reading emotional expressions from the face. However, most of the research to date has used static images and/or ‘extreme’ examples of facial expressions. Therefore, little is known about how expression intensity and dynamic stimuli might affect the commonly reported female advantage in facial emotion recognition. The current study investigated sex differences in accuracy of response (*H*_*u*_; unbiased hit rates) and response latencies for emotion recognition using short video stimuli (1sec) of 10 different facial emotion expressions (anger, disgust, fear, sadness, surprise, happiness, contempt, pride, embarrassment, neutral) across three variations in the intensity of the emotional expression (low, intermediate, high) in an adolescent and adult sample (*N* = 111; 51 male, 60 female) aged between 16 and 45 (*M* = 22.2, *SD* = 5.7). Overall, females showed more accurate facial emotion recognition compared to males and were faster in correctly recognising facial emotions. The female advantage in reading expressions from the faces of others was unaffected by expression intensity levels and emotion categories used in the study. The effects were specific to recognition of emotions, as males and females did not differ in the recognition of neutral faces. Together, the results showed a robust sex difference favouring females in facial emotion recognition using video stimuli of a wide range of emotions and expression intensity variations.

## Introduction

Facial expressions are an important means of communication [1], as they can carry inter-personal information, enabling promotion of bonding as well as the development and regulation of interpersonal relationships [2]. Facial expressions can be used to communicate emotional states to others and to regulate the environment by indicating people’s intentions and actions [3]. For example, an angry facial expression communicates dominance and rejection [4,5] facilitating avoidance by observers [6]. In contrast, a fearful face conveys submissiveness which facilitates approaching behaviour [6]. Thus the ability to recognise facial expressions of emotions helps to understand the emotional states, intentions, and behaviour of other people and plays an important role in everyday social interactions. There has been extensive research interest in facial expressions of emotion [7], especially on the ability to recognise facial expressions of emotion; a search in Google Scholar with the keyword ‘facial emotion recognition’ retrieved about 9,700 hits in April 2017.

One particular area of facial expression research that has received attention from the research community involves sex differences in facial emotion recognition. A number of meta-analyses and literature reviews have been published from this research area, which have generally reported that females outperform males in facial emotion recognition based on accuracy rates (e.g. [8–13]). Whereas the facial emotion recognition literature generally includes accuracy of response as the dependent variable (DV), response latencies are not included on a standard basis [14]. Nonetheless, some reports of females correctly recognising facial emotional expressions faster than males exist (e.g. [15–18]). Whereas much research on sex differences in facial emotion recognition have reported a female advantage over males when investigating sex differences in facial emotion recognition, some studies have not found a female advantage (e.g. [17,19]). It should be noted that small effect sizes are generally reported for this sex difference. For example, a meta-analysis carried out by Thompson and Voyer [13] reported a small effect size of Cohen’s *d* = 0.19 from the literature they included. It is possible that the underlying small effect size contributes to non-significant findings in some studies.

A common characteristic of most of the laboratory-based research on sex differences in facial emotion recognition to date is that static stimuli and/or stimuli portraying extreme examples of facial emotion have been used (e.g. [15,18,20–23]). However, facial expressions are dynamic by nature, and subtler facial expressions occur frequently in everyday social interactions [24]. Notably, subtler displays of facial emotional expressions are more difficult to recognise than more extreme (maximally intense) expressions, which affects accuracy and speed of recognition [22,25–28]. Consequently, it is important to investigate sex differences from dynamic emotion displays and across a range of expression intensities. This importance is emphasised given the female advantage in facial emotion recognition is generally reported as a small effect, and occasionally absent, in laboratory-based studies. It is also important to include accuracy of response and response latencies as DVs. Applying such methodology will allow for a more detailed analysis of the frequently reported female advantage in facial emotion recognition. Combining these aspects raises the question whether a female advantage in facial emotion recognition would be more clearly evident in more ecologically valid stimuli that are dynamic and include expression intensity variations.

One of the few studies investigating sex differences in facial emotion recognition and including expression intensity variations was conducted by JK Hall et al. [21]. These authors investigated sex differences in facial emotion recognition based on accuracy of response and response latencies. JK Hall et al. [21] included the six basic emotions of anger, disgust, fear, sadness, happiness, and surprise [29] and used expression intensity variations (ranging from 30% to 100%) within morphed static images. JK Hall et al. [21] reported a medium effect size for the female advantage in accuracy of response (*η*^2^ = .13) and for response latencies (*η*^2^ = .14), but no significant interaction of the factors ‘sex’ and ‘emotion category’. Since JK Hall et al. [21] averaged their data over the intensities for each emotion category, intensity level was not included as a factor in their analyses. Nontheless, it seems as though using more ecologically valid stimuli (i.e. expression intensity variations) retains the female advantage, possibly making it more pronounced.

To obtain information on sex differences in facial emotion recognition considering expression intensity, Hoffmann et al. [22] conducted two experiments. These authors investigated recognition of the six basic emotions plus contempt at varying intensities of facial emotional expression and ‘intensity’ was included as factor in their analysis. The varying intensities were created using a morphing technique, and presented to participants as static images. Hoffmann et al. [22] found females outperformed males, but only for recognition of the facial expressions in the intensity range of 40–70% of the full expressions, not for the intensity expressions in the range of 80–100%. The female advantage in recognition of the less intense facial emotional expressions aligns with reports of higher sensitivity in perception of facial emotional expressions in females compared to males [30]. Based on these reports, females seem to outperform males particularly when recognising less intense facial emotional expressions. This suggests that the small effect size for a female advantage in facial emotion recognition might be the result of the majority of research having used extreme facial expressions as stimuli. Another factor consistently reported to facilitate facial emotion recognition is motion (i.e. dynamic stimuli) [28,31]. This facilitation seems to be of particular importance for the recognition of subtler facial emotional expressions [32]. However, the literature including expression intensity variations thus far has used static stimuli. It is unknown how motion affects sex differences across expression intensity levels, which is particularly pertinent, as such stimuli have greater ecological validity than static images of extreme intensities.

Another study that included subtle and high expression intensity of facial emotion was conducted by Sasson et al. [33]. These authors used photographs of ‘re-lived’ emotional experiences of anger, fear, sadness, happiness, and neutral, and investigated accuracy of response in the typical manner (i.e. raw hit rates) as well as bias-corrected (i.e. unbiased hit rates). The investigation of unbiased hit rates is noteworthy, as the percentage of correct responses (raw hit rate) is corrected for biases or response habits. A bias can be the frequent misattribution of a specific emotion category to another emotion category. For example, always answering ‘anger’ when viewing both disgust and anger facial expressions. Such a bias would inflate the raw hit rates for anger despite a clear lack of discriminative ability from disgust. Unbiased hit rates factor in the misattributions. Examining unbiased hit rates thus provides information on facial emotion recognition abilities considering both sensitivity and specificity to individual emotion categories [34], i.e. recognising and discriminating emotions. To our knowledge, Sasson et al. [33] is the only published study on sex differences in facial emotion recognition including unbiased hit rates. For the raw hit rates, Sasson et al. [33] reported a female advantage throughout the expression intensities, and a greater female advantage for subtle than for intense expressions. This disproportionate advantage disappeared for the unbiased hit rates, with females outperforming males consistently across expression intensity levels based on unbiased hit rates. The differing findings regarding the female advantage seem to be the result of differences in response biases between the sexes at the lower expression intensities [33]. It is possible to speculate that the female advantage in raw hit rates was proportionally higher at low expression intensity than at high expression intensity due to a greater bias in females for a specific emotion at low expression intensity than at high expression intensity. That cue intensity is greater at high expression intensity than at low expression intensity would be an explanation for the bias occurring less frequently at high expression intensity. Combining these two aspects can explain the significant interaction of ‘sex’ and ‘expression intensity’ in the raw hit rates and the lack thereof in the unbiased hit rates in the study reported by Sasson et al. [33]. The importance of investigating unbiased hit rates is highlighted by the effect of response biases on the sex differences results in facial emotion recognition.

The aim of the current study was to compare samples of males and females on the recognition of facial emotion of varying intensity using video stimuli analysing unbiased accuracy rates and response latencies. This study adds to the limited knowledge about the role of expression intensity in facial emotion recognition and its impact on sex differences. This study extends the literature, as it is the first to investigate sex differences in facial emotion recognition based on unbiased hit rates from videos. In addition, the video set used in the current study includes expression intensity variations and a wider range of emotion categories than has been used in most previous research, including the six basic emotions, three complex emotions (contempt, embarrassment, pride), and neutral. The inclusion of a wider range of emotion categories and expression intensity variations aimed at increasing the ecological validity of the task, since more emotions than the basic emotions can be encountered in everyday life and they vary in intensity. It was hypothesised there would be a female advantage over males in facial emotion recognition based on both unbiased hit rates and response latencies. It was further hypothesised that the female advantage would be evident across all the intensity levels based on unbiased hit rates. The effect of emotional expression intensity on the speed of facial emotion recognition between the sexes based on response latencies was explored for the first time.

## Method

### Participants

There were 111 participants recruited for this study, which included current students and staff at the University of Bath, and prospective students, due to start at the University a short time later. The sample consisted of 51 male and 60 female participants aged between 16 and 45 (*M* = 22.2, *SD* = 5.7). Participants were recruited via electronic noticeboard, advertisements on noticeboards, word of mouth, and through Open Days held at the University of Bath. Participants aged under 18 were accompanied by a legal guardian, who gave permission for participation. All participants had normal or corrected-to-normal vision. None of the participants reported a clinical diagnosis of a mental disorder. The data of 104 participants of this sample has been used in previous analyses reported in Wingenbach et al. [27,28], but sex differences were not investigated in those reports. Ethical approval was given by the University of Bath Psychology Ethics Committee, and all participants gave written informed consent prior to participation.

### Face emotion stimuli

The Amsterdam Dynamic Facial Expression Set—Bath Intensity Variations (ADFES-BIV; [28]) was used as the video stimuli for presenting facial expressions of emotion, which was adapted from the ADFES stimulus set [35]. The ADFES-BIV comprises 360 experimental videos, plus an additional 10 videos used for practice trials. Each of the 10 emotion categories included in the video set includes 36 examples: expressed by 12 different encoders (7 male and 5 female) at three different expression intensities (low, intermediate, and high). Each video starts with a neutral blank stare and develops into one of these facial expressions: anger, disgust, fear, surprise, happiness, contempt, embarrassment, pride, sadness, or remains neutral. An example of the neutral expression at the start of the video and an example of low, intermediate, and high intensity disgust expression (based on the last frame of the videos) can be seen in Wingenbach et al. [28] (http://journals.plos.org/plosone/article?id=10.1371/journal.pone.0147112#sec002). The length of each of the videos is 1040ms, which includes 26 frames with a frame rate of 25/sec.

### Procedure

The testing sessions were conducted in a laboratory at the University of Bath with the experimenter present. After written consent was obtained, participants underwent the computer task which took approximately 35 minutes to complete. Participants were seated approximately 60cm from a 21-inch monitor. The computer task was identical to the task reported and validated in Wingenbach et al. [28]. The task started with an affective rating using the non-verbal Self-Assessment Manikin of valence and arousal (SAM; [36]) before and after a short neutral-content clip (4min 18sec) was presented. The neutral clip aimed to have all participants undergoing the facial emotion recognition task in a similar affective state to assure that group differences in affective states are not confounding the expected sex differences in facial emotion recognition. That the neutral clip effectively changes participants’ affective states is reported in Wingenbach et al. [28]. The facial emotion recognition labelling-task started with 10 practice trials (one for each emotion category) without providing feedback about the response, so participants could familiarise themselves with the task before the 360 experimental trials. Each trial started with a fixation cross presented for 500ms in the centre of the screen, followed by the stimulus with a resolution of 1024 x 768 (resembling the head size of direct face-to-face interactions), after which a blank screen was presented for 500ms before the answer screen appeared. The answer screen provided the 10 possible emotion terms to choose from by mouse click. The position of the emotion labels did not change, instead, the mouse position differed for each trial. Potential speed advantages in reaching certain labels were thereby accounted for. Participants were instructed to imagine actually interacting with the encoders and to respond intuitively, making immediate responses necessary. Response time was thus not restricted and missing values avoided. Participants were further instructed to keep the hand on the mouse. Since there were 10 answer choices for each trial, the chance level of response was 10%. Accuracy of response and response latency per trial were recorded. Participants were debriefed after the experiment, first year Psychology Undergraduate students received course credit and all other participants received £5 for participation.

### Data preparation and analysis

Males and females were compared regarding their valence and arousal ratings after watching a neutral clip to test if both groups were in a comparable affective state before the facial emotion recognition experiment. Since the variables were ordinal scaled and some of the data differed from normality, Mann-Whitney *U* tests were conducted for these group comparisons. Correlation coefficients (*r*) are presented as effect size measure using the formula *r* = *z*/sqrt(*N*) [37].

The DVs for the emotion recognition task were unbiased hit rates and response latencies. Wagner [38] has proposed a formula for *H*_*u*_ (i.e. unbiased hit rates) where the percentage of correct responses (raw hit rate) is corrected for response habits and biases. The formula takes into consideration both patterns of errors that can be seen in confusion matrices. That is, the misattribution of any emotion category to the target emotion, and the misattribution of a target emotion to any other emotion category. For example, for calculating the unbiased hit rates for ‘anger’, the correct identifications of anger are corrected by the misattribution of anger to any other emotion display and the misattribution of any other emotion to anger displays. The formula to calculate u*nbiased hit rate*s is: *H*_*u*_ = a^2^/(a + b + c) × (a + d + g), where *a* is the number of correct responses for a target emotion, *b* and *c* each the number of times a different emotion was labelled as the target emotion, and *d* and *g* the number of times the target emotion was labelled as a different emotion. The *H*_*u*_ formula produces percentages ranging from 0–100% and results are presented as such. *Response latency* was the time from the moment the answer screen was presented until the participant clicked the mouse on their answer choice. Response latency was measured in ms and differs from reaction time, as for the latter participants are instructed to answer as fast as possible. Only trials with correct responses were used in response latency analyses.

All DVs were inspected for extreme values using boxplots for males and females separately. Field [37] suggested the identified extreme values should be replaced by less extreme values. Consequently, the identified extreme values (+/- 3xIQR) were each changed to the lowest score on the respective variable within their group of sex, instead of eliminating the data. This did not change the rank of those cases, but made them less extreme.

Since the emotion category ‘neutral’ does not include different levels of intensities, it was not included in the main analyses. However, males and females were compared on the recognition of neutral faces to test for differences in the perception of faces. Since some of the data differed from normality, Mann-Whitney *U* tests were conducted to compare males and females on their unbiased hit rates of and response latencies to neutral faces.

Generalised linear mixed modelling (GLMM) was used to test the unbiased hit rates and response latency data. The model specifications were identical for both DVs, and included ‘subject’ and ‘sex’ as subject variables. The repeated statements were ‘intensity’ and ‘emotion’ due to their dependency characteristics. The fixed factors specified in the models were the main effects of ‘sex’, ‘emotion’, and ‘intensity’, as well as the interactions between all factors (‘sex*emotion’, ‘sex*intensity’, sex*emotion*intensity). The random intercept included was ‘subject’ to account for the variability of the intercepts between participants. The covariance structure specified was ‘diagonal’. Satterthwaite approximation for the degrees of freedom was applied due to the unbalanced design (*n*(males) = 51, *n*(females) = 60) and a robust estimation of the fixed effects and coefficients was applied to account for potential model violations. Contrasts were retrieved to follow up significant main effects; simple contrasts for the comparisons of males’ and females’ recognition, pairwise contrasts for comparisons of the intensity levels to each other, the emotions to each other, and the intensities within the emotions. Sequential Bonferroni-corrections for the contrasts to correct for multiple comparisons were applied. The results were hence compared to a *p*-value of .05 after sequential Bonferroni-correction; all *p*-values reported in the results section are after sequential Bonferroni-correction. For the unbiased hit rates, ANOVA specifications were applied, that is normal distribution with identity error function. Gamma distribution in combination with log error function was specified for the response latencies. The inclusion of correct trials only for the response latencies analysis resulted in a data reduction of 3.7%. Cohen’s *d* is presented as effect size measure for the primary results.

## Results

### Affective state comparison

Mann-Whitney *U* tests showed that males (*Mdn* = 2.00, *SD* = .92) did not differ significantly from females (*Mdn* = 2.00, *SD* = .74) on their post-neutral-clip *arousal* ratings (*U* = 1501.00, *z* = -.185, *p* = .853, r = .018). Males (*Mdn* = 3.00, *SD* = .76) also did not differ from females (*Mdn* = 3.00, *SD* = .70) on their post-neutral-clip *valence* ratings (*U* = 1545.00, *z* = .101, *p* = .920, *r* = .010). These results suggest that males and females underwent the facial emotion recognition task in comparable affective states. The male and female group were in a rather neutral affective state based on the group medians of low to medium arousal and neither positive nor negative valence.

### Neutral face recognition

Mann Whitney *U*-tests showed that males (*Mdn* = 55.68, *SD* = 17.12) did not differ significantly from females (*Mdn* = 66.23, *SD* = 16.34) in recognising neutral faces based on *unbiased hit rates* (*U* = 1750.00, *z* = 1.30, *p* = .193, *r* = .123). Mann Whitney *U*-tests showed no significant difference for recognition of neutral faces in males (*Mdn* = 669, *SD* = 166) compared to females (*Mdn* = 630, *SD* = 180) based on *response latencies* (*U* = 1359.00, *z* = -1.01, *p* = .312, *r* = .096). These results suggest that males and females did not differ in the general speed and accuracy of face processing based on recognition of neutral faces, which do not express emotions.

### Unbiased hit rates for dynamic emotion stimuli

**Fig 1:**
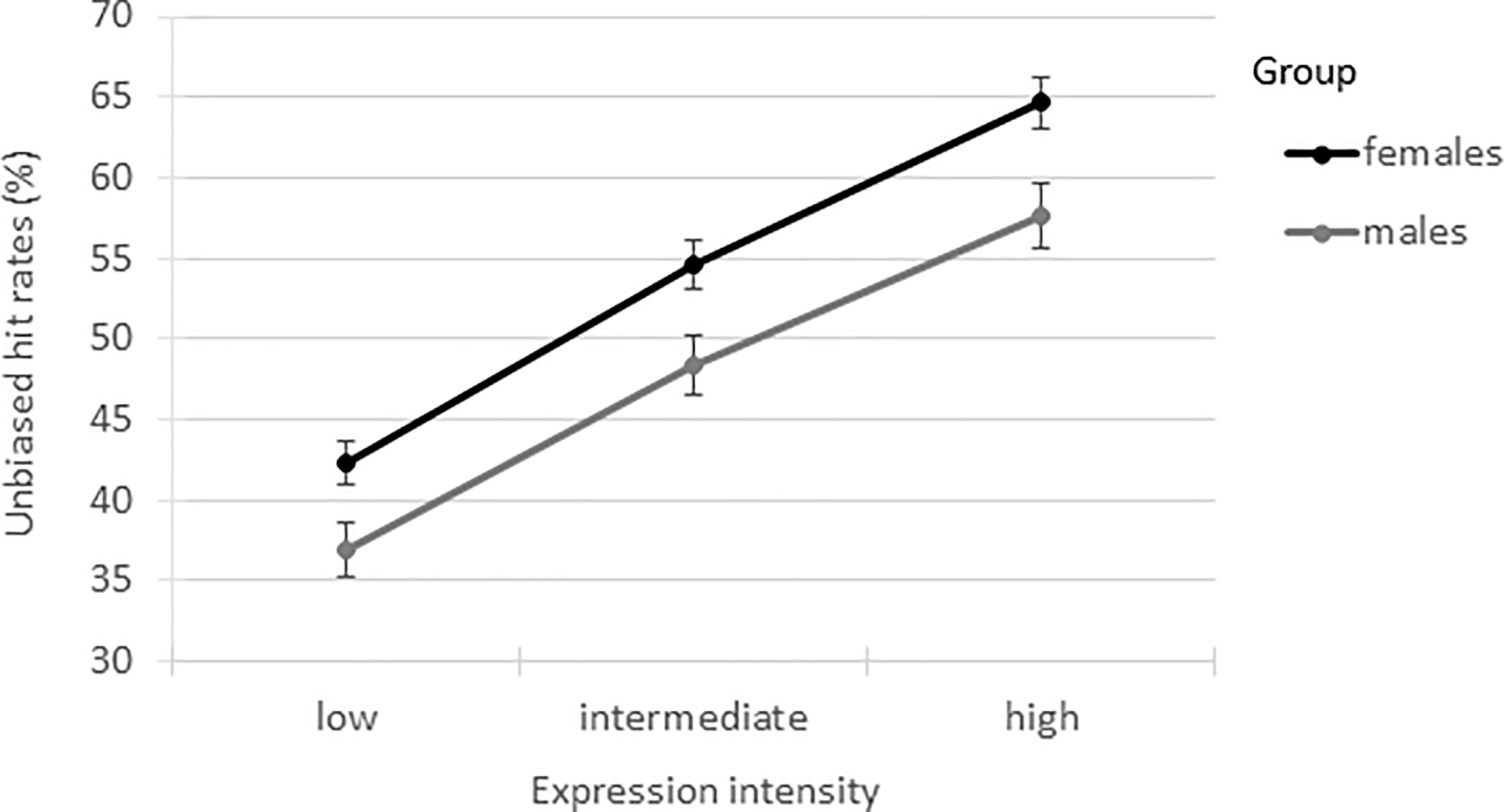
Unbiased hit rates (%) for males and females. Error bars represent the standard errors of the means.

The results from the GLMM for the unbiased hit rates showed a significant main effect of ‘sex’ (*F*(1,125) = 7.89, *p* = .006, Cohen’s *d* = 0.54) with females (*M* = 53.87, *SE* = 1.36, 95% CI [51.17, 56.58]) outperforming males (*M* = 47.64, *SE* = 1.75, 95% CI [44.17, 51.11]), as well as a significant main effect of ‘expression intensity’ (*F*(2,334) = 338.12, *p* < .001) with unbiased hit rates increasing with expression intensity level. The pairwise contrasts showed that the unbiased hit rates for the low intensity level (*M* = 39.62, *SE* = 1.07) were significantly lower (approximately 10%) than for the intermediate intensity level (*M* = 51.51, *SE* = 1.21; *t*(347) = -16.91, *p* < .001, 95% CI [-13.57, -10.19]), which in turn were significantly lower (approximately 10%) than for the high intensity level (*M* = 61.13, SE = 1.28; *t*(831) = -15.05, *p* < .001, 95% CI [-10.88, -8.37]). The interaction of ‘sex*intensity’ was not significant (*F*(2,334) = 0.50, *p* = .606); see Fig 1.

**Fig 2:**
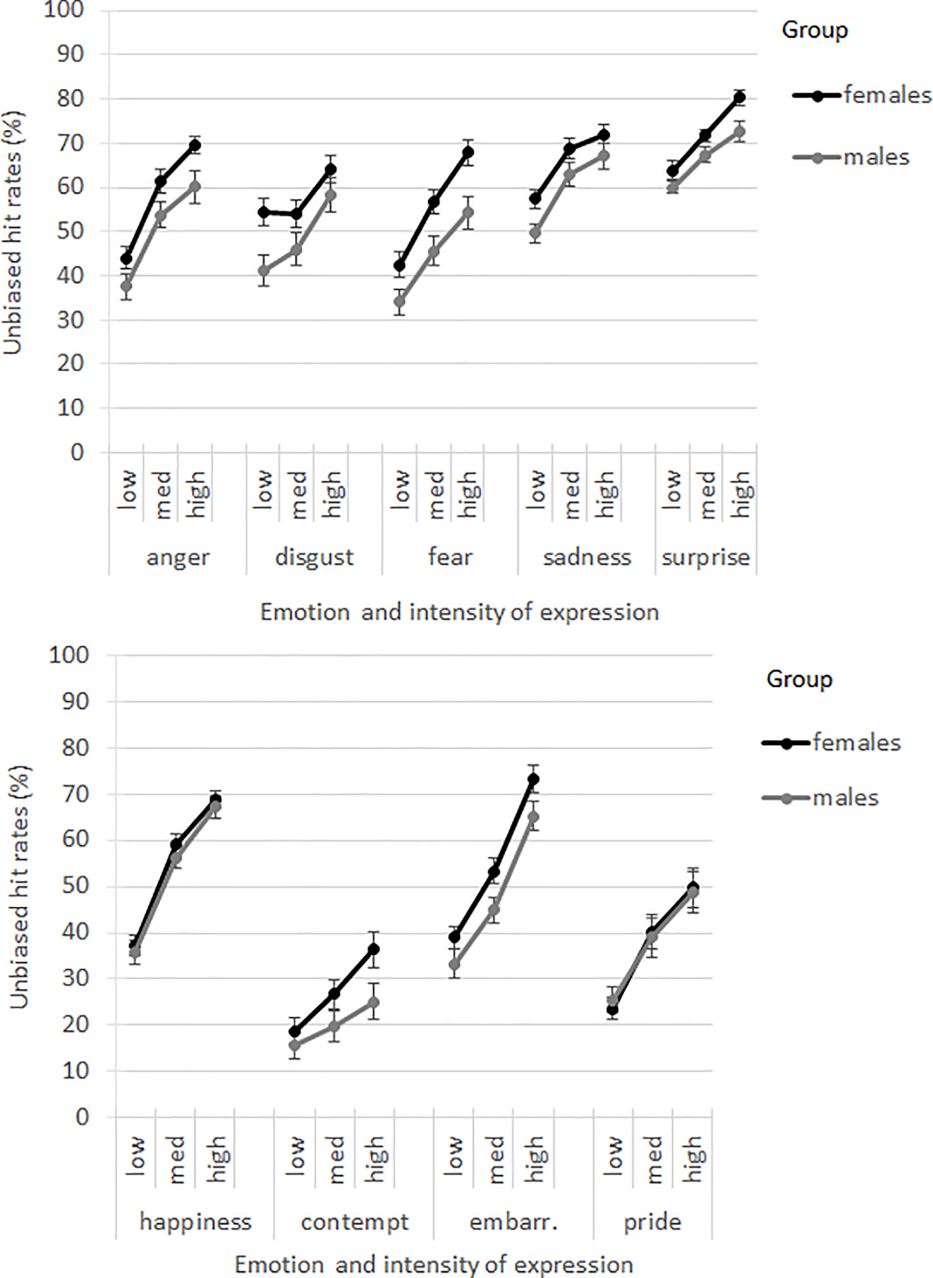
Unbiased hit rates (%) for males and females by emotion category and intensity level. Error bars represent the standard errors of the means.

The main effect of ‘emotion’ was significant (*F*(8,110) = 89.51, *p* < .001). The rank order of recognition from highest to lowest was: surprise, sadness, anger, happiness, disgust, embarrassment, fear, pride, and contempt; see Table 1. Pairwise contrasts showed that most emotions were significantly different from each other in unbiased hit rates (*p*’s ≤ .005). The emotions that did not differ significantly from each other in unbiased hit rates were anger, disgust, fear, happiness, and embarrassment (*p*’s > .557). The interaction of ‘emotion*intensity’ was significant (*F*(16,721) = 20.03, *p* < .001). For all emotions, the intensity levels differed from each other in unbiased hit rates (*p*’s ≤ .002), except for disgust where the unbiased hit rates were not significantly different between low and intermediate intensity (*p =* .181); see Table 1. The ‘sex*emotion’ (*F*(8,110) = 1.17, *p* = .321), and ‘sex*emotion*intensity’ interactions were not significant (*F*(16,721) = 1.40, *p* = .136); see Fig 2.

**Table 1:** Unbiased hit rates for each emotion category by intensity.

			Expression intensity					
Emotion (*N* = 111)		Total	low		intermediate		high	
	*M*	*SE*	*M*	*SE*	*M*	*SE*	*M*	*SE*
**Anger**	54.38	1.78	40.81	1.95	57.55	2.01	64.78	2.03
**Sadness**	62.94*	1.58	53.52	1.71	65.79	1.77	69.51	1.79
**Disgust**	53.00	2.22	47.82	2.41	49.94	2.37	61.24	2.64
**Fear**	50.16	1.92	38.27	2.08	51.15	2.10	61.07	2.34
**Happiness**	54.04	1.26	36.49	1.75	57.67	1.47	67.98	1.54
**Surprise**	69.29*	1.00	61.89	1.19	69.55	1.06	76.43	1.42
**Contempt**	23.74*	2.25	17.21	2.06	23.26	2.27	30.76	2.77
**Embarrassment**	51.43	1.64	36.11	1.95	49.05	1.89	69.14	2.15
**Pride**	37.81*	2.46	24.59	1.90	39.60	2.83	49.24	3.11

*Note*. Means (*M*) and standard errors of the means (*SE*) are expressed in percentages.

*These emotion categories were significantly different from each other, *p*’s ≤ .005.

### Response latencies for dynamic emotion stimuli

**Fig 3:**
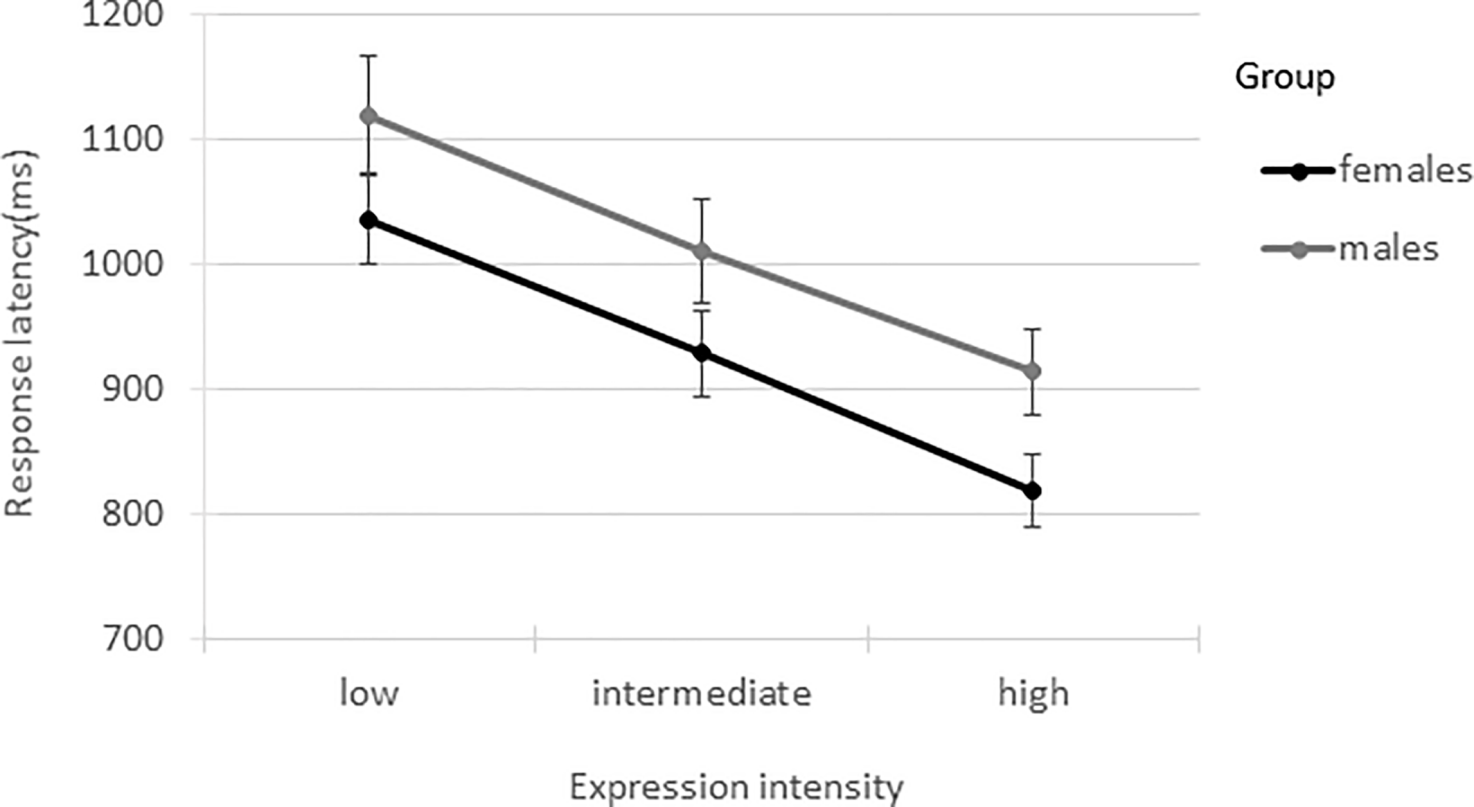
Overall response latencies (ms) for males and females. Error bars represent the standard errors of the means.

The main effect of ‘sex’ showed a trend towards significance (*F*(1,124) = 3.08, *p* = .082, Cohen’s *d* = 0.34) with females (*M* = 924, *SE* = 32, 95% CI [863, 989]) overall responding 88ms faster than males (*M* = 1012, *SE* = 39, 95% CI [937, 1092]) on average. The main effect of ‘expression intensity’ was significant (*F*(2,741) = 104.95, *p* < .001). Paired contrasts showed that participants were 108ms slower in recognising expressions at low intensity (*M* = 1077, *SE* = 30) than at intermediate intensity (*M* = 969, *SE* = 27, *t*(900) = 7.29, *p* < .001, 95% CI [79, 136]), and 104ms slower at recognising the intermediate intensity than the high intensity level (*M* = 865, *SE* = 23, *t*(532) = 7.57, *p* < .001, 95% CI [73, 135]). As with the unbiased hit rates, the ‘sex*intensity’ interaction was not significant (*F*(2,741) = 0.64, *p* = .527); see Fig 3.

The main effect of ‘emotion’ was significant (*F*(8,189) = 34.72, *p* < .001). The rank order of recognition, with a difference of 778ms between the emotion category fastest recognised and slowest recognised, was as follows: happiness, surprise, pride, sadness, disgust, anger, embarrassment, fear, contempt; see Table 2. Paired contrasts showed that most emotions were significantly different from each other in their response latencies (*t*’s(56–261) = 2.94–10.19, *p*’s < .014); response latencies to anger expressions were not significantly different from those to disgust expressions (*t*(131) = 0.63, *p* = 1.00), pride expressions (*t*(151) = -2.02, *p* = .336), and embarrassment expressions (*t*(124) = -0.77, *p* = 1.00), response latencies to disgust expressions were not significantly different from those to embarrassment expressions (*t*(177) = -1.39, *p* = .834), sadness expressions (*t*(133) = 2.05, *p* = .336), and pride expressions (*t*(127) = 2.03, *p* = .336), response latencies to happiness expressions were not significantly different from those to surprise expressions (*t*(159) = -0.57, *p* = 1.00) and pride expressions (*t*(127) = -2.24, *p* = .239), and response latencies to pride expressions were not significantly different from those to sadness expressions (*t*(177) = -0.45, *p* = 1.00) and surprise expressions (*t*(151) = -2.02, *p* = .336).

**Table 2:** Response times for each emotion category by intensity.

			Expression intensity					
Emotion (*N* = 111)	Total		low		intermediate		high	
	*M*	*SE*	*M*	*SE*	*M*	*SE*	*M*	*SE*
**Anger**	958^1^	32	1149	48	919	31	842	32
**Sadness**	877^2,4^	28	965	38	841	32	840	34
**Disgust**	938^1,2^	34	1017	45	989	43	821	30
**Fear**	1109	36	1259	49	1178	63	954	41
**Happiness**	790^3^	21	967	40	795	26	643	18
**Surprise**	801^3,4^	23	892	29	817	29	704	21
**Contempt**	1568	88	1417	82	1626	109	1673	130
**Embarrassment**	985^1,2^	36	1131	47	1020	48	828	36
**Pride**	863^1,2,3,4^	32	1028	55	780	35	801	36

*Note*. Means (*M*) and standard errors of the means (*SE*) are expressed in ms. Emotion categories sharing a superscript were not significantly different from each other, *p*’s > .05.

**Fig 4:**
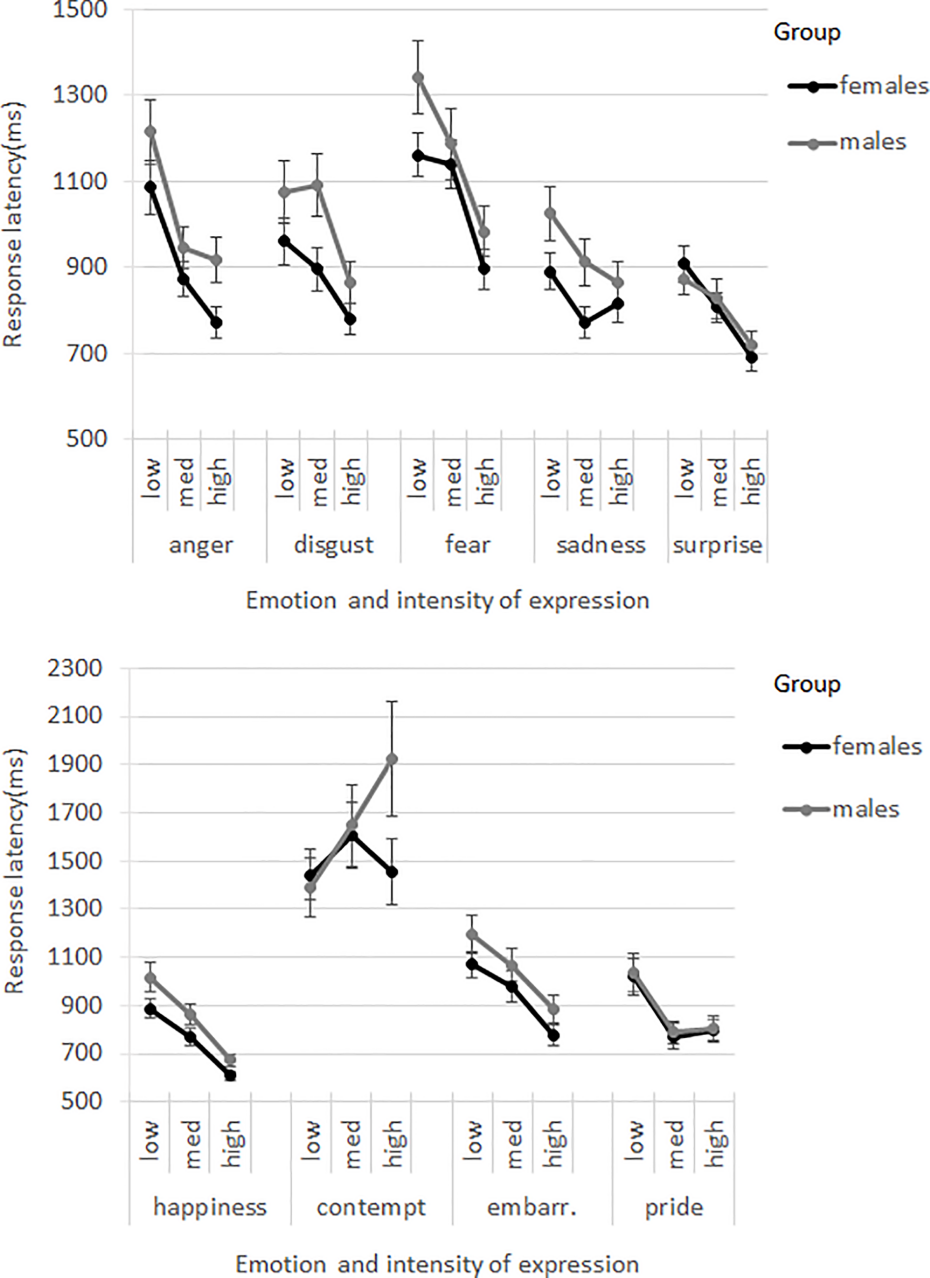
Response latencies (ms) for males and females by emotion category and intensity level. Error bars present the standard errors of the means.

The interaction ‘emotion*intensity’ was significant (*F*(16,501) = 9.55, *p* < .001). The response latencies decreased significantly with increasing intensity level for most emotion categories (*t*’s(82–243) = 2.29–10.10, *p*’s < .05); a trend towards a significant difference in response latencies was found between low and intermediate intensity expressions of contempt (*t*(227) = -2.08, *p* = .078). There were no significant differences in response latencies between the intermediate and high intensity levels for contempt (*t*(414) = -0.45, *p* = .654), between low and intermediate intensity disgust (*t*(338) = 0.76, *p* = .450), between low and intermediate intensity fear (*t*(139) = 1.69, *p* = .093), between intermediate and high intensity sadness (*t*(213) = 0.02, *p* = .988), and between intermediate and high intensity pride (*t*(290) = 0.57, *p* = .566); see Table 2. The interactions of ‘sex*emotion’ (*F*(8,189) = 1.37, *p* = .213) and ‘sex*emotion*intensity’ (*F*(16,424) = 1.34, *p* = .169) were not significant; see Fig 4.

## Discussion

This is the first study to report about sex differences in facial emotion recognition based on unbiased hit rates and also response latencies as well as having applied a methodology towards increasing ecological validity. The characteristics of facial emotional expressions as they are encountered in everyday life reflected in the stimuli used were: dynamic facial emotion (video recordings), varying expression intensities, and including a wide range of emotion categories (10 different emotions). Results showed a female advantage compared to males in facial emotion recognition based on unbiased hit rates; with a medium effect size. Results further showed a female advantage in recognising emotional expressions based on response latencies, though this was a trend towards significance, with a small to medium effect size. It is noteworthy that the sex differences were neither affected by levels of expression intensity nor emotion category included in the task. Findings revealed increasing accuracy (unbiased hit rates) and decreasing response latency with increasing level of expression intensity, with seemingly comparable increase and decrease respectively for males and females. Together, the results show a robust female advantage over males in recognising a wide range of emotional expressions from videos including intensity variations. These results illustrate a female superiority in facial emotion processing using dynamic and intensity-ranging stimuli that are more like real-life encounters (i.e. outside the laboratory).

The overall female advantage in facial emotion recognition found in the present study is consistent with the reported female advantage based on unbiased hit rates by Sasson et al. [33], who used static stimuli with two intensity levels. Notably, the present study extends previous findings to show that the female advantage is evident using dynamic stimuli (i.e. videos), across further intensity levels of expression, and a wider range of emotions. The results of the current study also align with previous findings showing that females recognise emotions from faces faster than males [15–18,39]. Being faster in recognising emotions in others would be beneficial during social interactions outside the laboratory, since facial expressions are naturally fleeting. A simple speed-accuracy trade-off of males responding faster causing more incorrect responses cannot explain the found sex differences. This is because males’ mean response latencies were not faster than females’ on any category included in the study. Further, the emotion categories with higher recognition rates were generally the emotion categories whereto faster correct identification occurred. In addition, the results of the current study showed that participants were more accurate and faster at recognition as the amount of emotional information displayed in the faces increased (i.e. with increasing expression intensity level). The sex differences did not differ across the three levels of expression intensity, showing that the female advantage was a robust effect. These results thus show that females are better at reading emotional facial expressions than males, no matter the degree of visual cues about the emotion displayed in the face. Since the female advantage did not rely on the degree of face muscle activation perceived in the faces of others, it suggests that the mechanisms for reading emotions are generally better in females compared to males.

That the factor ‘emotion’ did not influence the female advantage based on unbiased hit rates in the current study is not in line with the only other published report on sex differences based on unbiased hit rates. Sasson et al. [33] reported a female advantage that varied in extent across the four emotions included in their experiment. It should be noted that Sasson et al. [33] did not include two of the typical six emotion categories in their label-based facial emotion recognition experiment, surprise and disgust. However, these emotions are prone to lead to confusion with fear and anger respectively (e.g. [40–42]). Not including these emotion categories might have prevented the occurrence of these typical confusions and thus altered the results. Having included a wider range of emotion categories in the task of the present study allowed for more potential misattributions, which again has more ecological validity. Results showed that females seem to be better at recognising emotions from faces than males, independent of emotion category. This superiority could give females an advantage over males in various social situations outside the laboratory by allowing for appropriate behavioural responses related to the emotional expression observed (e.g. anger–resolution/avoidance).

A female advantage in response latencies in the current study is consistent with previous research [17,18,21]. It shows that females are faster than males at reading a wide number of face muscle activations that communicate specific emotions in others. Hampson et al. [15] reported the female advantage in response latencies was most prominent for negatively valenced emotions, although females also responded significantly faster to positively valenced emotions. The disparity in the female advantage reported by Hampson et al. [15] was based on combining individual emotions together for a comparison of positive vs negative valenced categories. This approach may have produced slightly different results to including individual emotion categories in the analysis as undertaken in the current study. Nonetheless, the female advantage in recognition speed is evident across valence and emotion categories. In the current study, females outperformed males on the recognition of basic as well as complex emotions, emotions that are easier and more difficult to recognise, as evidenced by the varying recognition rates for the individual emotion categories. The female advantage in facial emotion recognition (accuracy and speed) seems to become more general and robust when using stimuli of higher ecological validity. That is, stimuli including expression intensity variations of a wide range of emotions displayed in a dynamic manner. The current study thus demonstrates the importance to employ stimuli of high(er) ecological validity in future research.

The female superiority in facial emotion recognition found with the current study is further in line with single study reports and meta-analyses of sex differences in facial emotion recognition investigating *raw* hit rates (e.g. [8,10,12,16,20,21,23,33,43]). Previous research considering expression intensity in raw hit rates had suggested that females may have a greater advantage at lower intensity levels of emotion expression [22]. However, the current study’s findings are consistent with Sasson et al. [33], who suggest this disparity in advantage reflects sex differences in response biases (as discussed in the introduction). Therefore, it is important to include unbiased hit rates when investigating sex differences in facial emotion recognition. This importance is emphasised as sex differences in response biases when recognising emotions from others’ faces are evident earlier in life, by at least adolescence (see [16]). This aspect should be taken into consideration for future research.

One possibility for the current findings of sex differences in facial emotion recognition is that females simply have a better and faster ability to process faces in general, and that the female advantage in facial emotion recognition stems from this general face processing advantage. Hampson et al. [15] tested this idea and found the female advantage to be specific to facial emotion recognition. This is because no female advantage was found for face identity recognition. The current study similarly showed no significant female advantage for the recognition of neutral faces. In addition, females were found to generally be better at recognition of emotions from faces than males across both DVs in the task. Together, the results demonstrate that females outperform males specifically at facial *emotion* recognition. The female advantage is even evident when conscious processing of the emotional expressions is limited or prevented. Studies where exposure times to emotional face stimuli were sub-conscious (<200ms; [20]) or unconscious and automatic (33ms; [44]) have also reported a female advantage over males. Such reports imply that females outperform males in tasks requiring rapid processing of facial emotion. Consequently, Donges et al. [44] suggested that perceptual sensitivity might underlie the female advantage in facial emotion recognition. Hoffmann et al. [22] used exposure times to emotional content of 300ms and in the current study exposure time to emotional context was shorter than 1s (since all videos started with a neutral expression). Consequently, the female advantage at processing emotions from faces seems to extend to greater exposure times and conscious processing. It appears as though the female advantage in facial emotion recognition goes beyond a mere perceptual sensitivity, towards more accurate and faster processing of emotions from faces under various conditions. The resulting question is why females are better than males at recognising emotions from faces.

Evidence suggests that the differences between males and females in processing emotions might have a neural basis (see [45], for a review). For example, the right inferior frontal cortex is activated more in females and the left temporoparietal junction more in males during facial emotion recognition [46]. These differences may reflect different strategies used between the sexes when evaluating emotions [46] and could help explain sex differences in facial emotion recognition. An interesting postulation by Rahman et al. [17] is that males and females might have differing impressions as to when a happy or sad expression is present or in the perception of how an emotion is expressed. This postulation provides an alternative view to reports of sex differences in the objective capability to discriminate emotions (e.g. [23]). The postulation by Rahman et al. [17] fits in with reports by Lee et al. [47] that males and females differ in the areas of brain activation during emotion recognition of happy and sad facial expressions. This alternative view should gain attention in future research. To compare males’ and females’ imprints of emotion categories, reverse correlation of facial emotional expressions could be applied as conducted in Jack et al. [48].

The greater ability by females to correctly and rapidly recognise emotions could be tied to the primary importance of females for caretaker roles with children and within families. Being able to correctly and rapidly identify the emotion expressed by others would facilitate the understanding of emotional states during social interactions. This facilitation would allow for rapid responding to the needs of the others in an appropriate manner. For example, recognising sadness in another person can lead to comforting behaviour, which is important for bonding and nurturing roles. Fast and automatic processing of (facial) emotion might involve innate mechanisms designed through evolution to facilitate effective caring of offspring, as proposed by the primary caretaker hypothesis [49]. Conversely, the female recognition advantage might have been acquired as part of different emotional experiences and expectations that typically form females’ socialisation. According to the biosocial model [50], once a human is born, their biological sex determines the social labelling. This labelling leads to differential treatment of boys and girls. Generally, females are encouraged to display and pay attention to emotions and males get encouraged to suppress their emotional displays [51]. Thus, females may have an exposure advantage to facial emotional expressions compared to males. Derived from the reports by Calvo, Gutiérrez-García, Fernández-Martín, and Nummenmaa [52], this exposure advantage may lead to familiarisation that facilitates identification of facial emotional expressions. Calvo et al. [52] showed that we are better at recognising the emotions that we encounter most frequently in our social interactions, simply because we are familiar with them. It is therefore possible that females have developed their greater emotion processing abilities due to more exposure to emotional displays. It is further likely that biology and experience are interacting, leading to the female advantage in emotion processing including facial emotion recognition. It would thus be interesting to investigate gender and socialisation in relation to emotion recognition. For example, it could be investigated whether more gender-typical female identification and socialisation is associated with better recognition and male identification and socialisation with worse recognition.

The face emotion stimuli used in the current study were developed to include dynamic aspects and different degrees of intensity levels of facial emotional expressions that are similarly encountered in everyday life. However, the videos within the stimulus set are based on posed prototypical facial emotional expressions rather than naturalistic expressions of felt emotions elicited by particular situations. Although posed facial emotional expressions can be encountered in social interactions, future research should seek to replicate the current sex differences in facial emotion recognition using more spontaneous expressions resulting from emotion experience or live interactions to further increase ecological validity. The participant sample in the current study was also limited in terms of orientation towards a university-educated population and included predominantly young adults, which may limit the generalisability of the findings to the wider population.

In conclusion, this is the first study to identify a female advantage over males in facial emotion recognition based on unbiased hit rates and response latencies across varying expression intensities and a wide number of emotion categories, classified as basic and complex, from video stimuli.

## Supporting information

S1 Data Data from the valence and arousal ratings. (XLSX)

S2 Data Unbiased hit rates data. (XLSX)

S3 Data Response latencies data. (XLSX)
